# Bcl-2 resistant mitochondrial toxicity mediated by the isoquinoline carboxamide PK11195 involves de novo generation of reactive oxygen species

**DOI:** 10.1054/bjoc.2001.1788

**Published:** 2001-05

**Authors:** D A Fennell, M Corbo, A Pallaska, F E Cotter

**Affiliations:** Department of Experimental Haematology, St Bartholomew's & The Royal London School of Medicine, Turner Street, London E1 2AD, UK

**Keywords:** mitochondrial permeability transition, PK11195, reactive oxygen species, Bcl-2, anti-oxidants

## Abstract

Resistance to apoptosis is a major obstacle preventing effective therapy for malignancy. Mitochondria localized anti-death proteins of the Bcl-2 family play a central role in inhibiting apoptosis and therefore present valid targets for novel therapy. The peripheral benzodiazepine receptor (PBR) shares a close physical association with the permeability transition pore complex (PTPC), a pivotal regulator of cell death located at mitochondrial contact sites. In this study we investigated the cytotoxicity of the PBR ligand, PK11195, in the micromolar concentration range. PK11195 induced antioxidant inhibitable collapse of the inner mitochondrial membrane potential (ΔΨ_m_) and mitochondrial swelling in HL60 human leukaemia cells, but not in SUDHL4 lymphoma cells (which exhibited a higher level of reduced glutathione and relative tolerance to chemotherapy or pro-oxidant induced ΔΨ_m_ dissipation). PK11195 induced the production of hydrogen peroxide that was not inhibited by Bcl-2 transfection, nor depletion of mitochondrial DNA. ROS production was however blocked by protonophore, implicating a requirement for ΔΨ_m_. Our findings suggest that PK11195-induced cytotoxicity relies upon Bcl-2 resistant generation of oxidative stress; a process only observed at concentrations several orders of magnitude higher that required to saturate its receptor. © 2001 Cancer Research Campaign www.bjcancer.com


*British Journal of Cancer* (2001) **84**(10), 1397–1404
© 2001 Cancer Research Campaign
doi: 10.1054/ bjoc.2001.1788, available online at http://www.idealibrary.com on IDEAL®

http://www.bjcancer.com

### Bcl-2 resistant mitochondrial toxicity mediated by the isoquinoline carboxamide PK11195 involves de novo generation of reactive oxygen species

**DA Fennell, M Corbo, A Pallaska and FE Cotter**

Department of Experimental Haematology, St Bartholomew's & The Royal London School of Medicine, Turner Street, London E1 2AD, UK

**Summary** Resistance to apoptosis is a major obstacle preventing effective therapy for malignancy. Mitochondria localized anti-death proteins of the Bcl-2 family play a central role in inhibiting apoptosis and therefore present valid targets for novel therapy. The peripheral benzodiazepine receptor (PBR) shares a close physical association with the permeability transition pore complex (PTPC), a pivotal regulator of cell death located at mitochondrial contact sites. In this study we investigated the cytotoxicity of the PBR ligand, PK11195, in the micromolar concentration range. PK11195 induced antioxidant inhibitable collapse of the inner mitochondrial membrane potential ($\Delta\Psi_m$) and mitochondrial swelling in HL60 human leukaemia cells, but not in SUDHL4 lymphoma cells (which exhibited a higher level of reduced glutathione and relative tolerance to chemotherapy or pro-oxidant induced $\Delta\Psi_m$ dissipation). PK11195 induced the production of hydrogen peroxide that was not inhibited by Bcl-2 transfection, nor depletion of mitochondrial DNA. ROS production was however blocked by protonophore, implicating a requirement for $\Delta\Psi_m$. Our findings suggest that PK11195-induced cytotoxicity relies upon Bcl-2 resistant generation of oxidative stress; a process only observed at concentrations several orders of magnitude higher that required to saturate its receptor. © 2001 Cancer Research Campaign http://www.bjcancer.com

**Keywords:** mitochondrial permeability transition; PK11195; reactive oxygen species; Bcl-2; anti-oxidants

The peripheral benzodiazepine receptor (PBR) is an 18 000 Dalton transmembrane spanning protein (Joseph Liauzun et al, 1997) distinct from the central benzodiazepine receptor (Braestrup and Squires, 1977), that localizes to the outer mitochondrial membrane where it shares a close physical association with the voltage-dependent anion channel and adenine nucleotide translocator, constituents of the permeability transition pore complex (PTPC) (Anholt et al, 1986; McEnery et al, 1992). This multimeric structure mediates $Ca^{2+}$ and oxidative stress-inducible collapse of the inner mitochondrial membrane potential ($\Delta\Psi_m$) by forming a large non-specific megachannel with a size cutoff of around 1500 Daltons (Haworth and Hunter, 1979; Petronilli et al, 1994). Tremendous growth in interest in the physiology of the PTPC has arisen recently through its involvement in the regulation of both apoptotic and necrotic cell death, processes which involve early $\Delta\Psi_m$ dissipation (Lemasters et al, 1998). Furthermore, Bcl-2-related death agonist proteins (e.g. Bax, Bak) and antagonist proteins (e.g. Bcl-2, Bcl-$X_L$) have been shown to mediate their reciprocal regulatory actions on cell death directly via an interaction with the PTPC at the outer mitochondrial membrane (Narita et al, 1998; Shimizu et al, 1999).

The PBR specifically binds the compound 1-(2-chlorophenyl)-N-methyl-N-methyl-N-(1-methyl-propyl)-3 isoquinoline-carboxamide (PK11195), with nanomolar affinity. This ligand effects a diverse range of physiological actions including inhibition of cell proliferation and respiratory control (Hirsch et al, 1989; Camins et al, 1995b), effects on mitochondrial protein and cholesterol translocation (Papadopoulos et al, 1997; Wright and Reichenbecher, 1999), induction of cell differentiation (Landau et al, 1998), heat shock protein expression (Camins et al, 1995a), and facilitation of a diverse range of apoptotic stimuli (Pastorino et al, 1996; Hirsch et al, 1998; Verma et al, 1998). Consequently, a clearly defined physiological role for the PBR has remained elusive. PK11195 has been shown to reverse the chemoprotective effects of Bcl-2; an effect implicating PBR-mediated antagonism of Bcl-2, and promotion of megachannel formation by the PTPC (Hirsch et al, 1998). However, evidence from independent groups has suggested that the anti-proliferative and respiratory inhibiting effects of PK11195 are receptor-independent (Gorman et al, 1989; Camins et al, 1995b; Zisterer et al, 1998). Furthermore, the reported pro-apoptotic actions of PK11195 require concentrations several orders of magnitude higher than the reported nanomolar $K_D$ for the PBR (Le Fur et al, 1983). In this study, we have set out to investigate the cytotoxic effects of PK11195. Our findings suggest an important role for PK11195-induced oxidative stress in mediating cytotoxicity, and are consistent with a PBR independent mechanism of action.

*Received 11 September 2000*
*Revised 1 February 2001*
*Accepted 12 February 2001*

*Correspondence to:* DA Fennell

### MATERIALS AND METHODS

### Reagents

5,5′,6,6′-tetrachloro-1,1′,3,3′-tetraethylbenzimidazolocarbocyanine iodide (JC-1), 5-(and-6)-chloromethyl-2′,7′-dichlorodihydrofluorescein diacetate ($CMH_2DCFDA$), 5-chloromethylfluorescein diacetate (5-CMFDA), nonyl acridine orange, and 3-3′-dihexyloxacarbocyanine iodide ($DiOC_6(3)$) were purchased from Molecular Probes/Cambridge Bioscience, UK. N-acetylcysteine, VP16, ara-C, 5 N,N-dimethylamide (diamide), 1-(2-chlorophenyl)-N-

methyl-N-methyl-N-(1-methylpropyl)-isoquinoline carboxamide (PK11195), carbonylcyanide m-chlorophenylhdrazone (CCCP), propidium iodide and all cell culture reagents were purchased from Sigma-Aldrich Ltd, UK. FITC-conjugated mouse anti-human Bcl-2 monoclonal antibody, and the Dako intrastain fixation/permeabilization kit were purchased from Dako, UK.

### Cell culture and treatments

HL60, KG1A, K652 cells stably transfected with the Bcl-2 gene (K562 B4) or vector only (K562 N2) (kindly provided by Dr DE Banker and Dr F Applebaum, The Fred Hutchinson Cancer Research Center, USA), and SUDHL4 lymphoma cell lines, were maintained in exponential suspension cultures in RPMI 1640 medium supplemented with 10% fetal calf serum, 5 mM glutamine, 100 μg $ml^{-1}$ streptomycin and 100 U $ml^{-1}$ penicillin. Cells were grown in a humidified atmosphere of 5% $CO_2$/95% air at 37˚C. PK11195 was dissolved in ethanol at a stock concentration of 8.7 mg $ml^{-1}$, and added to cells at a 75 μM final concentration for 4 hours; vehicle alone was also used in medium. To test the effect of an antioxidant on PK11195 activity, cells were treated with 10 mM N-acetylcysteine for 45 minutes prior to treatment with PK11195.

Cells were incubated with VP16, or ara-C at 10 μM final concentration for 24 hours, or with the thiol crosslinking pro-oxidant, diamide, at 100 μM concentration for 24 hours. The protonophore CCCP was used to dissipate $\Delta\Psi_m$ by treating cells at a concentration of 10 μM for 15 minutes prior to treatment with PK11195.

### Depletion of mitochondrial DNA

KG1A cells were depleted of mitochondrial DNA by culturing for 4 months in medium supplemented with ethidium bromide (400 ng $ml^{-1}$), uridine (5 μg $ml^{-1}$), and glucose (4.5 mg $ml^{-1}$). Loss of mitochondrial DNA was confirmed by 15 cycles of PCR, amplifying a 1023 base pair fragment from the D loop region (upstream primer 5′ CAC CAT TAG CAC CCA AAG CT 3′, reverse primer 5′ CTG TTA AAA GTG CAT ACC GCC A 3′). Semi-quantitative PCR was performed on equal concentrations and serial dilutions of $\rho^0$ and $\rho^+$ DNA, and normalized against nuclear DNA.

### Mitochondrial isolation

Cells were homogenized mechanically in buffer A (250 mM sucrose, 10 mM tris, and 1 mM EGTA, pH 7.8 at 4˚C), and the homogenate centrifuged at 600 ***g*** for 10 minutes to remove membranes and nuclei. This was followed by a second spin at 15 000 ***g*** for 5 minutes in buffer B (250 mM sucrose, 10 mM tris) to obtain a mitochondrial pellet. Analysis at single mitochondrial resolution was performed by flow cytometry using a FACScan (Becton Dickinson, UK), running cellquest software; 30 000 events were aquired in listmode and the mitochondrial population isolated for analysis using a forward versus orthogonal light scatter gate.

### Measurement of $\Delta\Psi_m$ and mitochondrial mass at single cell resolution

Cells were labelled with 5 μg $ml^{-1}$ of the delocalized lipophilic cation JC-1 for 10 minutes at 37˚C in the dark, prior to analysis by flow cytometry. In the presence of a negative membrane potential, JC-1 forms aggregates which fluoresce in the orange wavelength to provide a measure of $\Delta\Psi_m$, whereas the monomeric form fluoresces in the green wavelength providing a measure of mitochondrial mass. The FL-1 (green) and FL-2 (orange) fluorescence signals corresponding to the forward and orthogonal scatter gate circumscribing 10 000 cells were acquired with logarithmic amplification. To determine the proportion of cells with intact plasma membrane undergoing $\Delta\Psi_m$ in response to chemotherapy and diamide, cells were incubated for 20 minutes at 37˚C in the dark with $DiOC_6(3)$ (80 nM), then counter stained for 10 minutes with propidium iodide (20 μg $ml^{-1}$). Events with increased PI fluorescence (FL3, red) were removed by gating from the $DiOC_6(3)$ histograms (FL1) to eliminate dead cells.

### Measurement of hydrogen peroxide generation and reduced glutathione

Cells were loaded for 15 minutes at 37˚C in the dark with $CMH_2DCFDA$ (5 μM) prior to treatment with PK11195, and washed once prior to analysis by flow cytometry using the FL1 fluorescence detector. Cellular reduced glutathione (GSH) was estimated by loading cells with 1 μM 5-CMFDA for 45 minutes at 37˚C in the dark. This probe bonds covalently with intracellular sulfhydryl groups (predominantly GSH) and is then deacetylated to yield the fluorescent product 5-CMF. Cells were washed once prior to flow cytometry and 5-CMF fluorescence was detected in the FL1 wavelength.

### Flow cytometric estimation of Bcl-2 oncoprotein expression

Cells ($10^6$) were pelleted and fixed for 15 minutes in the dark using the Dako intrastain solution A (100 μl). The fixed cells were then simultaneously permeabilized and labelled with 1 μg of FITC labelled Bcl-2 antibody for 15 minutes in Dako intrastain solution B. Cells were washed, and analysed using the FL1 detector.

### Statistics

Unpaired Student's *t*-test was used to evaluate the statistical differences between experiments repeated in triplicate. *P* values shown are two-sided.

### RESULTS

### PK11195 mediates anti-oxidant inhibitable $\Delta\Psi_m$ collapse in HL60 cells but not SUDHL4 cells

The effect of PK11195 on $\Delta\Psi_m$ was investigated in a range of cell lines using the mitochondrial potentiometric probe JC-1 (Reers et al, 1995). HL60 leukaemia cells treated with PK11195 exhibited a reduction in JC-1 aggregate fluorescence consistent with a collapse in $\Delta\Psi_m$ (Figures 1A and 1B). Pre-treatment of HL60 cells with 10 mM N-acetylcysteine, a sulfhydryl donor and antioxidant, prevented the collapse in $\Delta\Psi_m$ mediated by PK11195 (Figure 1C). PK11195 failed to dissipate $\Delta\Psi_m$ in the Bcl-2 hyperexpressing SUDHL4 lymphoma cell line, but rather, consistently mediated an increase in JC-1 green fluorescence resulting in the appearance of a second, discrete population of cells with decreased JC-1 orange:JC-1 green fluorescence ratio (Figures 1D and 1E).

 © 2001 Cancer Research Campaign

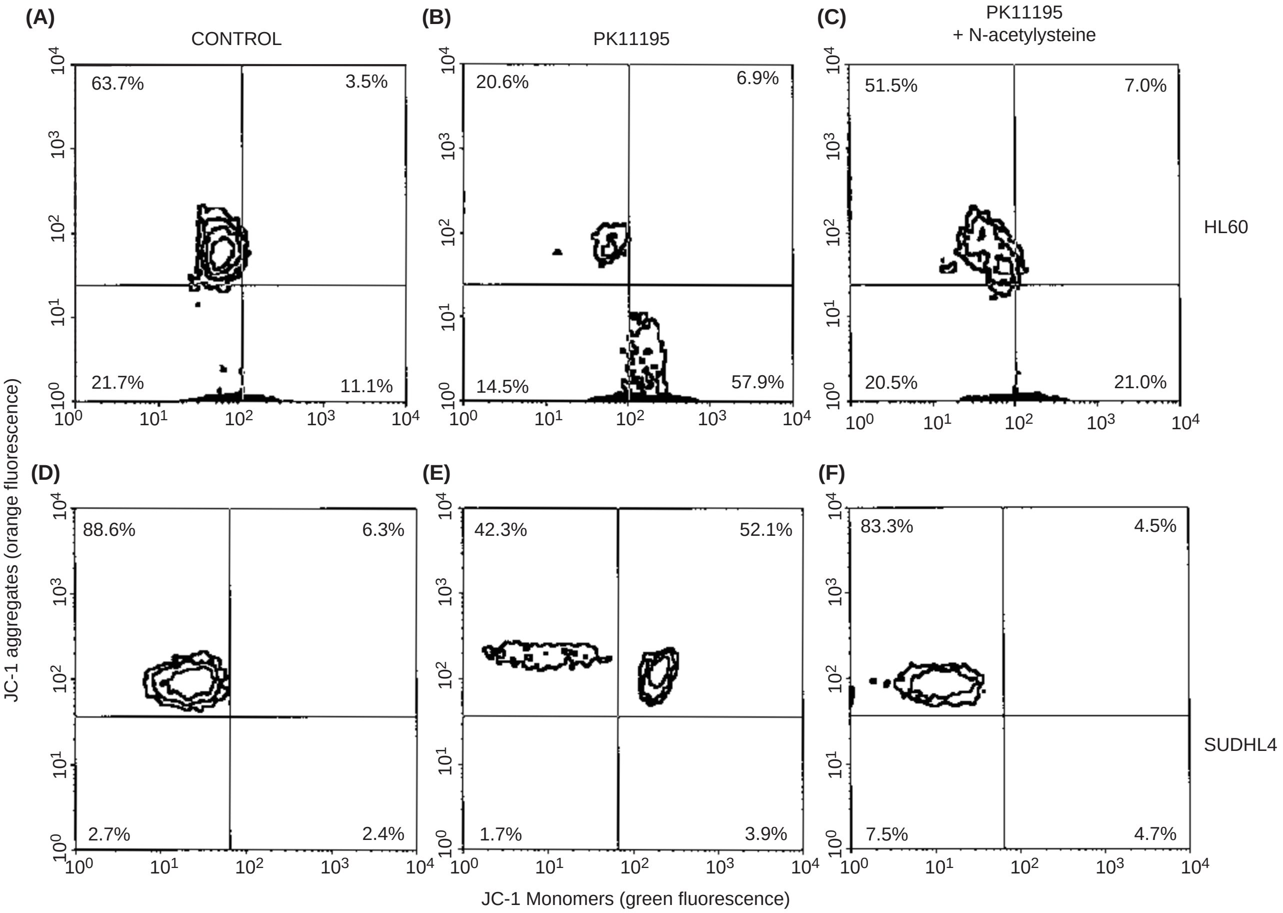

**Figure 1** Differential effects of PK11195 on $\Delta\Psi_m$ in HL60 versus SUDHL4 cells. (**A**) HL60 cells stained with the potentiometric probe JC-1 exhibit orange aggregate fluorescence reflecting mitochondrial energization (y-axis), and green monomer fluorescence reflecting mitochondrial mass (x-axis). Control cells are localized to the upper left quadrant. (**B**) Treatment with PK11195 for 4 hours reduced JC-1 orange fluorescence, dissipating $\Delta\Psi_m$, and leading to an increased frequency of events in the lower right quadrant. (**C**) Pre-treatment with 10 mM N-acetylcysteine prevented PK11195 induced $\Delta\Psi_m$ collapse. (**D**) SUDHL4 cells stained with JC-1 (control) localize to the upper left quandrant. (**E**) Treatment of SUDHL4 cells with PK11195 resulted in the emergence of a population with high JC-1 green fluorescence localizing to the upper right quadrant. (**F**) Pre-treatment of SUDHL4 cells with 10 mM N-acetylcysteine prevented the PK11195 mediated increase in JC-1 green fluorescence

Changes in JC-1 fluorescence in SUDHL4 cells were temporally associated with an increase in the intensity of mitochondrial cardiolipin labelling by nonyl-acridine orange. The appearance of the PK11195-induced SUDHL4 cell population exhibiting an increase in JC-1 green fluorescence was inhibited by 10 mM N-acetylcysteine (Figure 1F).

The differential sensitivity of HL60 versus SUDHL4 cells to PK11195 induced $\Delta\Psi_m$ collapse was also reflected in the differential tolerance of these cell lines to mitochondrial depolarization induced by cytotoxic chemotherapy and the pro-oxidant diamide, as measured by the potentiometric probe $DiOC_6(3)$. Treatment with 10 µM Ara-C or 10 µM VP16 mediated $\Delta\Psi_m$ collapse in a greater fraction of HL60 cells compared with SUDHL4 cells (Figure 2A); this order of differential sensitivity was also observed after treatment with 100 µM diamide (Figure 2A).

### PK11195 induces generation of reactive oxygen species independently of a functional respiratory chain

To investigate the differential effects of PK11195 on HL60 and SUDHL4 cells, the production of reactive oxygen species (ROS) was measured at single cell resolution using the fluorescent probe $CMH_2DCFDA$ which covalently bonds to intracellular sulfhydryl groups during loading, and is de-acetylated by hydrogen peroxide to generate the fluorescent product $CMH_2DCF$. Following treatment with PK11195, an increase in green fluorescence of greater than one log, was observed in both cell lines (Figure 2B and 2C).

To investigate the role of the respiratory chain in mediating the production of ROS, KG1A leukaemia cells were depleted of their mitochondrial DNA (KG1A $\rho^0$) by long-term culture in the presence of ethidium bromide (Figure 2D). Both $\rho^+$ and $\rho^0$ KG1A cells responded to treatment with PK11195 by increasing $CMH_2DCF$ fluorescence (Figures 2E and 2F).

### Bcl-2 expression does not prevent PK11195-induced ROS generation

To determine the effect of Bcl-2 on PK11195-induced production of ROS, K562 myeloid leukaemia cells transfected with the *Bcl-2* gene (Figure 3A) were treated with PK11195 after loading with $CMH_2DCFDA$. Bcl-2 expression, shown by immunofluorescence to be predominantly mitochondrial, (Dr Deborah Banker, personal communication), did not affect the PK11195-induced increase in $CMH_2DCF$ fluorescence, which was equivalent in K562 N2 and K562 B4 cell lines (Figures 3B and 3C).

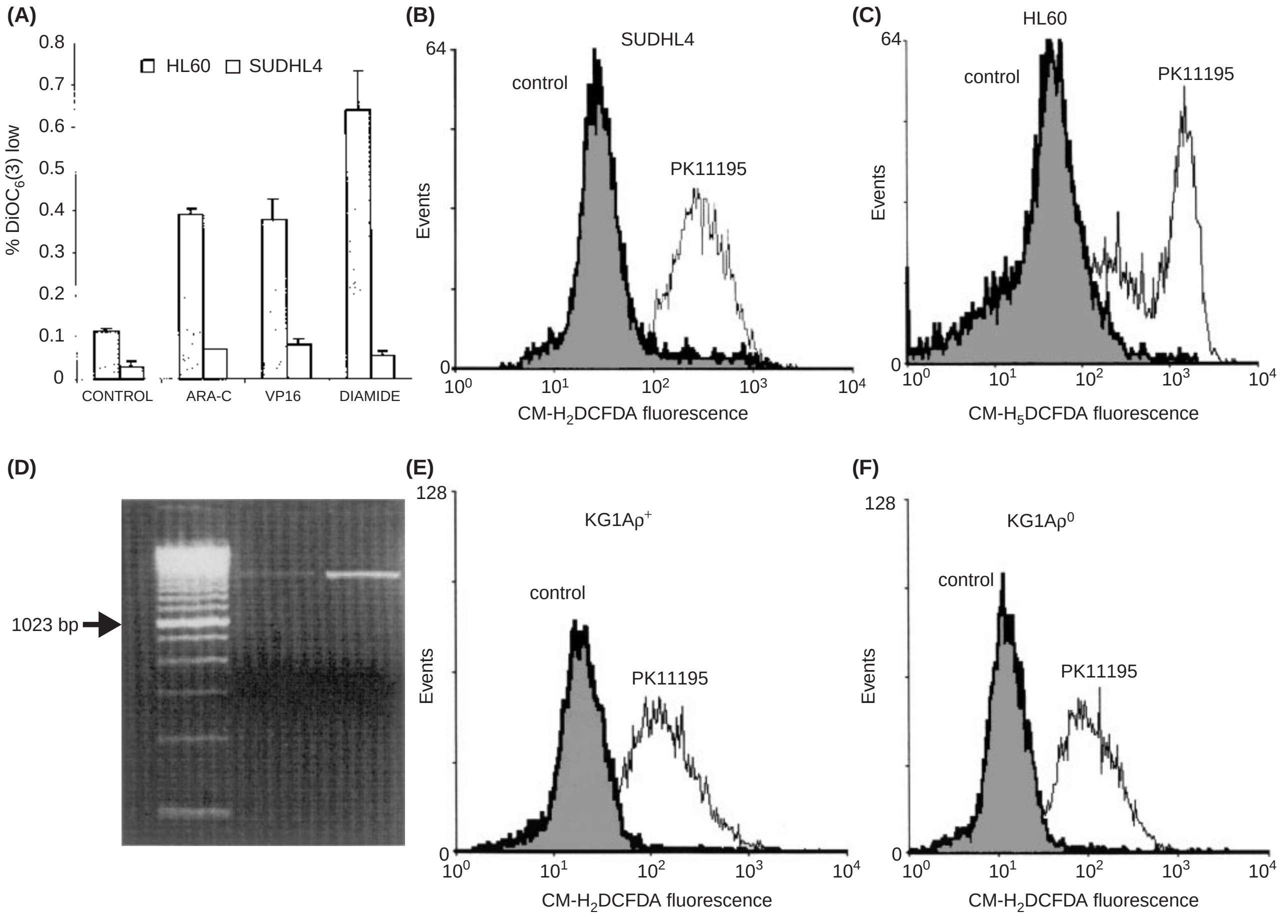

**Figure 2** PK11195 mediates ROS production in HL60, SUDHL4 cells and $\rho^0$ cells. (**A**) HL60 cells exhibited relative sensitivity to $\Delta\Psi_m$ collapse measured by $DiOC_6(3)$, compared to SUDHL4 cells, induced by the cytotoxic chemotherapeutic drugs ara-c and VP16, as well as the pro-oxidant diamide. (**B**) PK11195 induced ROS production in SUDHL4 cells with a one log fold increase in $CMH_2DCF$ fluorescence. (**C**) PK11195 induced ROS generation was associated with a two log fold increase in $CMH_2DCF$ fluorescence. (**D**) Loss of mitochondrial DNA in $\rho^0$ cells demonstrated by PCR amplification of a 1023 basepair amplicon from the D loop region. (**E**) KG1A $\rho^+$ cells responded to PK11195 by generating ROS. F. PK11195 generated ROS in KG1A $\rho^0$ cells

The differential effects of the pro-oxidant diamide on HL60 and SUDHL4 cells, and the reversibility of PK11195 induced $\Delta\Psi_m$ collapse in HL60 cells by antioxidant, suggested that the underlying sensitivity of these contrasting cell lines may result from intrinsic differences in their oxidant stress buffering capacities. Using the sulfhydryl-reactive fluorescent probe 5-CMFDA to estimate reduced glutathione (GSH) at single cell resolution, Bcl-2 transfection was found not to produce a change in 5-CMF fluorescence (Figure 3D). However, SUDHL4 cells exhibited greater 5-CMF fluorescence compared with HL60 cells (Figure 3E).

### Mitochondrial toxicity mediated by PK11195 requires mitochondrial energization

To examine the pro-oxidant effects of PK11195 at the sub-cellular level, mitochondria were isolated from HL60 and SUDHL4 cells after treatment with PK11195. HL60 mitochondria consistently increased the median of their forward light scatter distribution by approximately one log compared with control, consistent with an increase in mitochondrial size occurring at a time coincident with ROS generation (Figure 3F). However, an increase in forward scatter was not observed in SUDHL4 mitochondria after 4 hours of incubation (not shown).

Production of ROS by PK11195 was found to be restricted to cells with normal (non-apoptotic) morphology, reflected in the forward and side light scatter distributions (FSC and SSC respectively). Cells undergoing apoptosis exhibit an increase in SSC (reflecting an increased granularity), and a reduction in FSC (reflecting a reduction in cell size); we have previously observed an association with $\Delta\Psi_m$ collapse in this low FSC/high SSC population (Fennell and Cotter, 2000). HL60 cells treated with PK11195 exhibited an increase in apoptotic (low FSC/high SSC) frequency associated with a reduction in membrane potential (Figure 4A), that was not seen in SUDHL4 cells. When the apoptotic and normal cells were gated (using the regions denoted R1 and R2 respectively), ROS production was only observed in the morphologically normal cells (Figure 4B). This observation suggested a requirement for mitochondrial energization in the generation of ROS by PK11195. To test this hypothesis, HL60 cells were treated with the protonophore CCCP to collapse $\Delta\Psi_m$ prior to treatment with PK11195 (Figure 4C). In the presence of CCCP, PK11195 failed to increase $CMH_2DCF$ fluorescence compared with control, in contrast to cells treated with PK11195 in the absence of the protonophore (Figures 4D and 4E).

### DISCUSSION

In this study, we have investigated the cytotoxicity of the PBR ligand PK11195 in a range of haemopoetic cell lines. The relative susceptibility of HL60 cells versus SUDHL4 cells to mitochondrial

 © 2001 Cancer Research Campaign

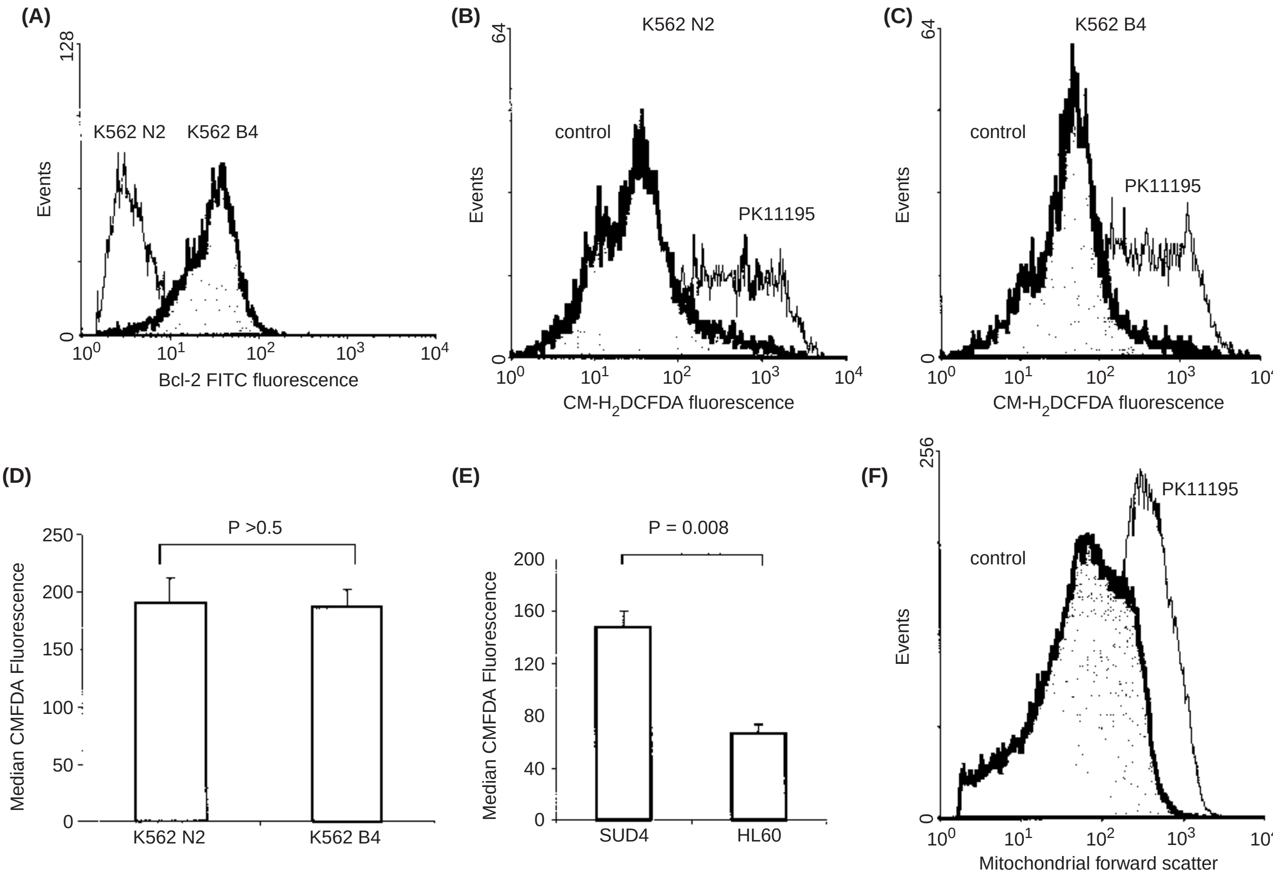

**Figure 3** ROS production by PK11195 is Bcl-2 resistant and is temporally associated with an increase in mitochondrial size. (**A**) K562 B4 cells transfected with the Bcl-2 gene exhibited increased Bcl-2 oncoprotein expression compared with K562 N2 cells, as measured by immunofluorescence. (**B**) K562 N2 responded to PK11195 treatment by increasing $CMH_2DCF$ fluorescence. (**C**) Bcl-2 transfection did not prevent PK11195 induced ROS generation in K562 B4 cells. (**D**) Reduced cellular glutathione content measured at single cell resolution by CMFDA fluorescence was not affected by Bcl-2 transfection in K562 B4 versus K562 N2 cells. (**E**) SUDHL4 cells exhibited a greater reduced glutathione (GSH) content compared with HL60 cells. (**F**) HL60 mitochondria isolated from PK11195-treated cells exhibited an increase in the distribution of forward scatter reflecting a general increase in individual size depolarization induced by PK11195, chemotherapeutic drugs and the pro-oxidant diamide suggests a role for intrinsic anti-oxidant defences in modulating the toxicity of PK11195. This was supported by the observation that exogenous antioxidant treatment protects HL60 cells from PK11195 induced mitochondrial toxicity, consistent with an involvement of ROS in mediating $\Delta\Psi_m$ collapse. The ability of PK11195 to induce ROS generation in the presence of sustained $\Delta\Psi_m$ in SUDHL4 cells implies that $\Delta\Psi_m$ collapse is not a prerequisite for ROS generation, in contrast to the paradigm described by Zamzami et al in relation to several other apoptosis-inducing toxins (Zamzami et al, 1995).

SUDHL4 cells exhibited a relatively higher GSH concentration compared with HL60 cells and could account for the greater intensity of PK11195-induced $CMH_2DCF$ fluorescence in HL60 cells (approximately one log fold). Bcl-2 transfection was not found to attenuate the generation of ROS in K562 cells, nor modulate cellular GSH content; however, Bcl-2 could conceivably antagonize ROS-induced permeability transition via its well described stabilizing effect on the permeability transition pore complex, the gating of which is regulated by the redox state of critical vicinal thiols (Costantini et al, 1996, 2000; Marzo et al, 1998). This would be consistent with our finding that PK11195 can induce mitochondrial swelling in HL60 mitochondria, but not in SUDHL4 mitochondria which hyperexpress Bcl-2 due to t(14;18).

The increase in JC-1 monomer fluorescence that we observed in PK11195 treated SUDHL4 cells has been previously reported to occur as an early event during some forms of apoptosis, and has been shown to reflect mitochondrial proliferation using corroborative transmission electron microscopy (TEM) (Mancini et al, 1997, 1998). The observed increase in cardiolipin labelling of SUDHL4 cells by nonyl-acridine orange has also been reported by Miccoli et al to occur in PK11195-treated human glioma cells, consistent with an increase in mitochondrial mass (Miccoli et al, 1999). Becker's group reported PK11195-induced mitochondrial proliferation using TEM in rat C6 glioma cells and pituitary $GH_3$ cells (Shiraishi et al, 1991; Black et al, 1994), however, the basis for this proliferation was not investigated. Transcription of nuclear mitochondrial biogenesis genes is induced by oxidative stress (Miranda et al, 1999), consistent with observations by us and by Camins et al (Camins et al, 1995a) that PK11195 induces ROS, and may therefore underlie the antioxidant reversible increase in JC-1 green and NAO fluorescence.

PK11195 facilitated mitochondrial permeability transition induced by a diversity of apoptosis inducers has been previously

*© 2001 Cancer Research Campaign*

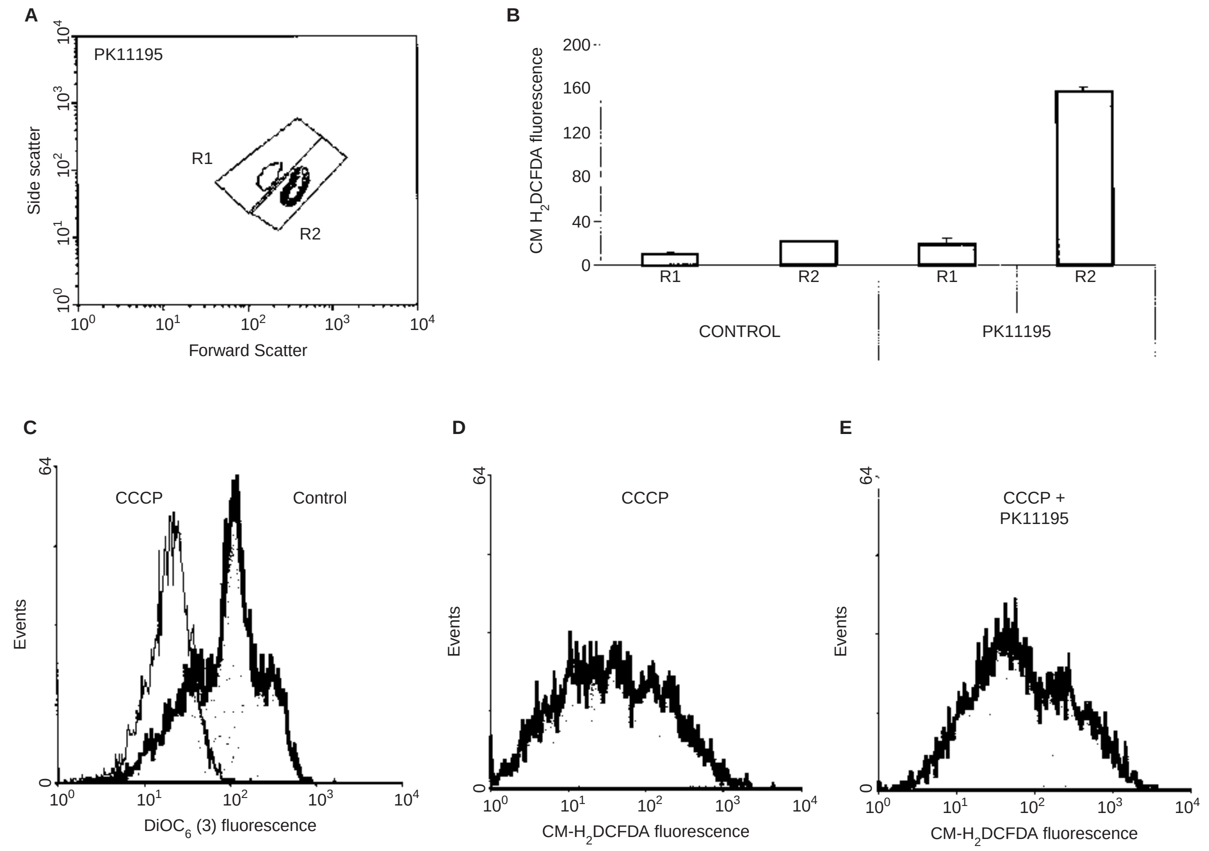

**Figure 4** Requirement for mitochondrial energisation in the production of ROS by PK11195 in HL60 cells. (**A**) Distribution of forward and side light scatter of PK11195 treated HL60 cells with regional gating of apoptotic (R1) and normal (R2) subpopulations. (**B**) Histogram showing the relative increase in $CMH_2DCF$ fluorescence associated with PK11195 treatment in HL60 cells from gates R1 and R2 morphology. (**C**) CCCP (10 μM) induced reduction in $\Delta\Psi_m$ in HL60 cells measured as a lower DiOC$_6$(3) fluorescence. (**D**). $CMH_2DCF$ fluorescence in HL60 cells treated with 10 μM CCCP only (control) (**E**). PK11195 failed to increase $CMH_2DCF$ fluorescence in HL60 cells treated with 10 μM CCCP

reported (Pastorino et al, 1994, 1996; Hirsch et al, 1998), as well as direct induction of $\Delta\Psi_m$ dissipation in thymocytes (Tanimoto et al, 1999). The requirement for micromolar concentrations of PK11195 to achieve the effects described in this study suggests a mechanism of toxicity that is independent of the PBR, since the equilibrium-binding constant is only 1 nM (Le Fur et al, 1983). Carayon et al showed that PBR transfection could protect Jurkat leukaemia cells from ROS induced cytotoxicity (Carayon et al, 1996). However, we have observed that SUDHL4 and BV173 cells exhibit comparable PBR expression, (measured using 7-nitro-2,1,3-benzoxadiazol-4-y1 derivative of 2-phenylindole-3-acetamide (NBD FGIN-1-27 analogue), a fluorescent PBR probe (Kozikowski et al, 1997)), yet exhibit differential tolerance to the effects of PK11195 on $\Delta\Psi_m$; furthermore, Jurkat T cells which lack demonstrable NBD FGIN-1-27 binding, dramatically produce ROS in response to PK11195 (unpublished data). The origin of PK11195 induced ROS is unknown. $CMH_2DCFDA$ is oxidized to $CMH_2DCF$ by $H_2O_2$, which is generated intracellularly by dismutation of superoxide catalysed by manganese superoxide dismutase (MnSOD). The principal mitochondrial defence against $H_2O_2$ is the glutathione redox cycle system which converts $H_2O_2$ to $H_2O$ at the expense of GSH oxidation to its disulfide, GSSG, by the action of glutathione peroxidase. The relatively lower 5-CMF fluorescence of HL60 cells, is consistent with a lower total cellular GSH level, which could increase susceptibility to oxidation of the mitochondrial GSH pool by PK11195 compared with SUDHL4 cells. Disruption of mitochondrial $H_2O_2$ defences allows the generation of hydroxyl radical via contact with transition metal ions ($Cu^+$ or $Fe^{2+}$) with the potential to trigger permeability transition. The chloromethyl function of $CMH_2DCFDA$ is thiol reactive, however, GSH is present in the millimolar range (some 3 orders of magnitude greater than the concentration of $CMH_2DCFDA$), arguing on stoichiometric grounds, against an artefactual pertubation in intracellular GSH levels as a basis for PK11195-mediated ROS.

Generation of $H_2O_2$ in KG1A $\rho^0$ cells suggests that a functional respiratory chain is not required for PK11195-induced ROS. However, we have shown in this study that $\Delta\Psi_m$ (maintained in $\rho^0$ cells by reverse proton flux through complex V) is a pre-requisite for ROS production. PK11195 may therefore require $\Delta\Psi_m$-dependent entry into the mitochondrial matrix in a manner resembling the isoquinoline derivative N-methyl 4-phenyl pyridine, which occurs after protonation of 1 methyl 4 phenyl 1,2,3,6 tetrahydropyridine (MPTP) (Ramsay et al, 1986); experiments to test this electrophoretic import hypothesis are currently under investigation. The reported Bcl-2 reversing efficacy of PK11195 suggests that this compound may possess useful apoptosis-sensitizing efficacy, with potential application for the therapy of apoptosis-resistant malignancies (Hirsch et al, 1998). Further investigations to unravel underlying mechanisms of its pro-apop-

 *© 2001 Cancer Research Campaign*

totic activity should help to separate PBR versus PBR ligand-dependent processes, and in so doing, clarify the biological role of this elusive receptor.

### ACKNOWLEDGEMENTS

We thank the Medical Research Council (DAF was supported by a Medical Research Council Clinical Training Fellowship), and Dr Martin Grootveld for stimulating discussions.

### REFERENCES

Anholt RRH, Pedersen PL, De Souza EB and Snyder SH (1986) The peripheral-type benzodiazepine receptor. Localization to the mitochondrial outer membrane. *J Biol Chem* **261**: 576–583

Black KL, Shiraishi T, Ikezak K, Tabuchi K and Becker DP (1994) Peripheral benzodiazepine stimulates secretion of growth hormone and mitochondrial proliferation in pituitary tumour GH3 cells. *Neurol Res* **16**: 74–80

Braestrup C and Squires RF (1977) Specific benzodiazepine receptors in rat brain characterized by high-affinity (3H) diazepam binding. *Proc Natl Acad Sci USA* **74**: 3805–3809

Camins A, Diez-Fernandez C, Camarasa J and Escubedo E (1995a) Cell surface expression of heat shock proteins in dog neutrophils induced by mitochondrial benzodiazepine receptor ligands. *Immunopharmacology* **29**: 159–66

Camins A, Diez-Fernandez C, Pujadas E, Camarasa J and Escubedo E (1995b) A new aspect of the antiproliferative action of peripheral-type benzodiazepine receptor ligands. *Eur J Pharmacol* **272**: 289–292

Carayon P, Portier M, Dussossoy D, Bord A, Petitpretre G, Canat X, Le Fur G and Casellas P (1996) Involvement of peripheral benzodiazepine receptors in the protection of hematopoietic cells against oxygen radical damage. *Blood* **87**: 3170–3178

Costantini P, Chernyak BV, Petronilli V and Bernardi P (1996) Modulation of the mitochondrial permeability transition pore by pyridine nucleotides and dithiol oxidation at two separate sites. *J Biol Chem* **271**: 6746–6751

Costantini P, Belzacq AS, Vieira HL, Larochette N, de Pablo MA, Zamzami N, Susin SA, Brenner C and Kroemer G (2000) Oxidation of a critical thiol residue of the adenine nucleotide translocator enforces bcl-2-independent permeability transition pore opening and apoptosis [In Process Citation]. *Oncogene* **19**: 307–314

Fennell DA and Cotter FE (2000) Stochastic modeling of apoptosis tolerance distributions measured by multivariate flow analysis of human leukemia cells. *Cytometry* **39**: 266–274

Gorman AM, O'Beirne GB, Regan CM and Williams DC (1989) Antiproliferative action of benzodiazepines in cultured brain cells is not mediated through the peripheral-type benzodiazepine acceptor. *Biol Psychol* **28**: 265–269

Haworth RA and Hunter DR (1979) The $Ca^{2+}$-induced membrane transition in mitochondria. II. Nature of the Ca2+ trigger site. *Arch Biochem Biophys* **195**: 460–467

Hirsch JD, Beyer CF, Malkowitz L, Beer B and Blume AJ (1989) Mitochondrial benzodiazepine receptors mediate inhibition of mitochondrial respiratory control. *Mol Pharmacol* **35**: 157–163

Hirsch T, Decaudin D, Susin SA, Marchetti P, Larochette N, Resche-Rigon M and Kroemer G (1998) PK11195, a ligand of the mitochondrial benzodiazepine receptor, facilitates the induction of apoptosis and reverses Bcl-2-mediated cytoprotection. *Exp Cell Res* **241**: 426–434

Joseph Liauzun E, Farges R, Delmas P, Ferrara P and Loison G (1997) The M(r) 18,000 subunit of the peripheral-type benzodiazepine receptor exhibits both benzodiazepine and isoquinoline carboxamide binding sites in the absence of the voltage-dependent anion channel or of the adenine nucleotide carrier. *J Biol Chem* **272**: 28102–28106

Kozikowski AP, Kotoula M, Ma D, Boujrad N, Tuckmantel W and Papadopoulos V (1997) Synthesis and biology of a 7-nitro-2,1,3-benzoxadiazol-4-yl derivative of 2-phenylindole-3-acetamide: a fluorescent probe for the peripheral-type benzodiazepine receptor. *J Med Chem* **40**: 2435–2439

Landau M, Weizman A, Zoref-Shani E, Beery E, Wasseman L, Landau O, Gavish M, Brenner S and Nordenberg J (1998) Antiproliferative and differentiating effects of benzodiazepine receptor ligands on B16 melanoma cells. *Biochem Pharmacol* **56**: 1029–1034

Le Fur G, Perrier ML, Vaucher N, Imbault F, Flamier A, Benavides J, Uzan A, Renault C, Dubroeucq MC and Gueremy C (1983) Peripheral benzodiazepine binding sites: effect of PK 11195, 1-(2-chlorophenyl)-N-methyl-N-(1-methylpropyl)-3-isoquinolinecarboxamide. I. In vitro studies. *Life Sci* **32**: 1849–1856

Lemasters JJ, Nieminen AL, Qian T, Trost LC, Elmore SP, Nishimura Y, Crowe RA, Cascio WE, Bradham CA, Brenner DA and Herman B (1998) The mitochondrial permeability transition in cell death: A common mechanism in necrosis, apoptosis and autophagy. *Biochim Biophys Acta Bioenerg* **2**: 177–196

Mancini M, Anderson BO, Caldwell E, Sedghinasab M, Paty PB and Hockenbery DM (1997) Mitochondrial proliferation and paradoxical membrane depolarization during terminal differentiation and apoptosis in a human colon carcinoma cell line. *J Cell Biol* **138**: 449–469

Mancini M, Sedghinasab M, Knowlton K, Tam A, Hockenbery D and Anderson BO (1998) Flow cytometric measurement of mitochondrial mass and function: a novel method for assessing chemoresistance. *Ann Surg Oncol* **5**: 287–295

Marzo I, Brenner C, Zamzami N, Susin SA, Beutner G, Brdiczka D, Remy R, Xie ZH, Reed JC and Kroemer G (1998) The permeability transition pore complex: A target for apoptosis regulation by caspases and Bcl-2-related proteins. *J Exp Med* **187**: 1261–1271

McEnery MW, Snowman AM, Trifiletti RR and Snyder SH (1992) Isolation of the mitochondrial benzodiazepine receptor: association with the voltage-dependent anion channel and the adenine nucleotide carrier. *Proc Natl Acad Sci USA* **89**: 3170–3174

Miccoli L, Oudard S, Beurdeley-Thomas A, Dutrillaux B and Poupon MF (1999) Effect of 1-(2-chlorophenyl)-N-methyl-N-(1-methylpropyl)-3-isoquinoline carboxamide (PK11195), a specific ligand of the peripheral benzodiazepine receptor, on the lipid fluidity of mitochondria in human glioma cells [In Process Citation]. *Biochem Pharmacol* **58**: 715–721

Miranda S, Foncea R, Guerrero J and Leighton F (1999) Oxidative stress and upregulation of mitochondrial biogenesis genes in mitochondrial DNA-depleted HeLa cells. *Biochem Biophys Res Commun* **258**: 44–49

Narita M, Shimizu S, Ito T, Chittenden T, Lutz RJ, Matsuda H and Tsujimoto Y (1998) Bax interacts with the permeability transition pore to induce permeability transition and cytochrome c release in isolated mitochondria. *Proc Natl Acad Sci USA* **95**: 14681–14686

Papadopoulos V, Amri H, Boujrad N, Cascio C, Culty M, Garnier M, Hardwick M, Li H, Vidic B, Brown AS, Reversa JL, Bernassau JM and Drieu K (1997) Peripheral benzodiazepine receptor in cholesterol transport and steroidogenesis. *Steroids* **62**: 21–28

Pastorino JG, Simbula G, Gilfor E, Hoek JB and Farber JL (1994) Protoporphyrin IX, an endogenous ligand of the peripheral benzodiazepine receptor, potentiates induction of the mitochondrial permeability transition and the killing of cultured hepatocytes by rotenone. *J Biol Chem* **269**: 31041–31046

Pastorino JG, Simbula G, Yamamoto K, Glascott PA, Jr, Rothman RJ and Farber JL (1996) The cytotoxicity of tumor necrosis factor depends on induction of the mitochondrial permeability transition. *J Biol Chem* **271**: 29792–29798

Petronilli V, Costantini P, Scorrano L, Colonna R, Passamonti S and Bernardi P (1994) The voltage sensor of the mitochondrial permeability transition pore is tuned by the oxidation-reduction state of vicinal thiols. Increase of the gating potential by oxidants and its reversal by reducing agents. *J Biol Chem* **269**: 16638–16642

Ramsay RR, Dadgar J, Trevor A and Singer TP (1986) Energy-driven uptake of N-methyl-4-phenylpyridine by brain mitochondria mediates the neurotoxicity of MPTP. *J Biochem Toxicol* **1**: 13–29

Reers M, Smiley ST, Mottola-Hartshorn C, Chen A, Lin M and Chen LB (1995) Mitochondrial membrane potential monitored by JC-1 dye. *Methods Enzymol* **260**: 406–417

Shimizu S, Narita M and Tsujimoto Y (1999) Bcl-2 family proteins regulate the release of apoptogenic cytochrome c by the mitochondrial channel VDAC [see comments]. *Nature* **399**: 483–487

Shiraishi T, Black KL, Ikezaki K and Becker DP (1991) Peripheral benzodiazepine induces morphological changes and proliferation of mitochondria in glioma cells. *J Neurosci Res* **30**: 463–474

Tanimoto Y, Onishi Y, Sato Y and Kizaki H (1999) Benzodiazepine receptor agonists modulate thymocyte apoptosis through reduction of the mitochondrial transmembrane potential. *Jpn J Pharmacol* **79**: 177–183

Verma A, Facchina SL, Hirsch DJ, Song SY, Dillahey LF, Williams JR and Snyder SH (1998) Photodynamic tumor therapy: mitochondrial benzodiazepine receptors as a therapeutic target. *Mol Med* **4**: 40–45

Wright G and Reichenbecher V (1999) The effects of superoxide and the peripheral benzodiazepine receptor ligands on the mitochondrial processing of manganese-dependent superoxide dismutase. *Exp Cell Res* **246**: 443–450

Zamzami N, Marchetti P, Castedo M, Decaudin D, Macho A, Hirsch T, Susin SA, Petit PX, Mignotte B and Kroemer G (1995) Sequential reduction of

© 2001 Cancer Research Campaign mitochondrial transmembrane potential and generation of reactive oxygen species in early programmed cell death. *J Exp Med* **182**: 367–377
Zisterer DM, Hance N, Campiani G, Garofalo A, Nacci V and Williams DC (1998) Antiproliferative action of pyrrolobenzoxazepine derivatives in cultured cells: absence of correlation with binding to the peripheral-type benzodiazepine binding site. *Biochem Pharmacol* **55**: 397–403

 *© 2001 Cancer Research Campaign*
